# Relations between gross motor skills and executive functions, controlling for the role of information processing and lapses of attention in 8-10 year old children

**DOI:** 10.1371/journal.pone.0224219

**Published:** 2019-10-24

**Authors:** Irene M. J. van der Fels, Joanne Smith, Anne G. M. de Bruijn, Roel J. Bosker, Marsh Königs, Jaap Oosterlaan, Chris Visscher, Esther Hartman

**Affiliations:** 1 University of Groningen, University Medical Center Groningen, Center for Human Movement Sciences, Groningen, The Netherlands; 2 University of Groningen, Groningen Institute for Educational Research, Groningen, The Netherlands; 3 University of Groningen, Faculty of Behavioral and Social Sciences, Department of Educational Sciences, Groningen, The Netherlands; 4 Emma Children’s Hospital, Amsterdam UMC, University of Amsterdam and Vrije Universiteit Amsterdam, Emma Neuroscience Group, Department of Pediatrics, Amsterdam Reproduction & Development, Amsterdam, The Netherlands; 5 Vrije Universiteit Amsterdam, Clinical Neuropsychology Section, Amsterdam, The Netherlands; 6 VU University Medical Center, Department of Pediatrics, Amsterdam, The Netherlands; The Education University of Hong Kong, HONG KONG

## Abstract

This study aimed to systematically investigate the relation between gross motor skills and aspects of executive functioning (i.e. verbal working memory, visuospatial working memory, response inhibition and interference control) in 8–10 year old children. Additionally, the role of information processing (speed and variability) and lapses of attention in the relation between gross motor skills and executive functions was investigated. Data of 732 Dutch children from grade 3 and 4 were analyzed (50.0% boys, 50.4% grade 3, age = 9.16 ± 0.64 years). Gross motor skills were assessed using three items of the Körper Koordinationstest für Kinder and one item of the Bruininks-Oseretsky test of Motor Proficiency, Second Edition. Executive functions were assessed using the Wechsler Digit Span task (verbal working memory), the Visuospatial Memory task (visuospatial working memory), the Stop Signal task (response inhibition) and a modified version of the Flanker task (interference control). Information processing and lapses of attention were obtained by applying an ex-Gaussian analysis on go trials of the Stop Signal task. Multilevel regression analysis showed that gross motor skills were significantly related to verbal working memory, visuospatial working memory and response inhibition, but not to interference control. Lapses of attention was a significant predictor for all executive functions, whereas processing speed was not. Variability in processing speed was only predictive for visuospatial working memory. After controlling for information processing and lapses of attention, gross motor skills were only significantly related to visuospatial working memory and response inhibition. The results suggest that after controlling for information processing and lapses of attention, gross motor skills are related to aspects of executive functions that are most directly involved in, and share common underlying processes with, gross motor skills.

## Introduction

There is a growing body of research investigating the relation between gross motor skills and executive functions in children. Different explanations for these relations between the motor and cognitive domains exist. From a neuropsychological view, it is proposed that there is overlap in neural networks that are important for gross motor skills and for executive functions, thereby explaining relations between the two domains [1]. At a behavioral level, there may be certain types of executive functions that are more relevant for, and therefore may be stronger related to, gross motor tasks [2]. However, due to the small number of studies focusing on specific executive functions and inconsistent results of the few studies that did examine specific executive functions, further research is needed to investigate relations between gross motor skills and specific aspects of executive functions [3]. There are also indications that information processing and attention are closely involved in the relation between gross motor skills and executive functions [4,5]. However, the influence of these variables on the relation between gross motor skills and executive functions has not yet been investigated together in typically developing children. The current study aims to fill these gaps by investigating relations between gross motor skills and specific aspects of executive functions. Secondly, the role of information processing and attention will be examined. The insights gained by this study will help our understanding of the specificity of relations between gross motor skills and executive functions, which can give useful handles for several practical applications.

### Gross motor skills and executive functions

Gross motor skills represent the involvement of large body muscles in balance, limb, and trunk movements [6]. The gross motor skills that children obtain and develop during childhood form the foundation for the later development of more complex movements and sport-specific skills. Therefore, gross motor skills are strong predictors for a life-long active lifestyle [7]. Besides being important for physical development, gross motor skills are also important for the development of executive functions. Children are involved in physical activities that require goal-directed behavior, thereby supporting the development of executive functions [8].

Executive functions refer to cognitive processes that are involved in purposeful, goal-directed behavior [8,9]. These functions play a critical role in children’s development, as they have shown to be strongly related to academic achievement, and are seen as vital for success throughout life [10–13]. Two of the core aspects of executive functions are working memory and inhibition [14,15]. Working memory is understood as the ability to store and manipulate information in short-term memory, whereby specialized processes exist for verbal and visual information [16]. Inhibition is the ability to deliberately suppress dominant, automatic, or prepotent responses, and conflicting stimuli [17,18]. Within the inhibition factor, a further distinction can be made between response inhibition and interference control. Response inhibition refers to the ability to suppress planned actions that are no longer required or inappropriate [17], whereas interference control refers to the ability to cognitively suppress conflicting stimuli [18]. These specific executive functions are related to each other, but are also clearly separable [15]. The specificity of these executive functions indicates that some of them may be more relevant for gross motor tasks than others.

Cross-sectional behavioral studies have shown that gross motor skills are related to visuospatial working memory [19], while contradictory findings have been reported for verbal working memory [19,20]. In addition, positive relations have been shown between gross motor skills and interference control [2,19,20], but not between gross motor skills and response inhibition [2]. Ludyga et al. [21] showed that different aspects of gross motor skills were differently related to working memory and response inhibition; locomotor skills were related to working memory, whereas object control was related to response inhibition. In line with these cross-sectional findings, interventions aiming to improve motor skills have also been shown to enhance specific aspects of executive functions. Koutsandreou et al. [22] showed that a 10-week motor intervention improved working memory in 9–10 year old children. Alesi et al. [23] found positive effects of a soccer intervention on motor skills and specific aspects of working memory. The intervention did enhance visuospatial working memory, whereas there was no effect on verbal working memory. Pesce et al. [24] found that a physical activity intervention for six months improved motor skills (manual dexterity, ball skills, and balance) and inhibition, but there was no effect on working memory. The specificity of the findings in those studies are difficult to explain, because little is known about the specific relations between gross motor skills and executive functions.

### Explanations for relations between gross motor skills and executive functions

The relation between gross motor skills and executive functions is often explained by an overlap in brain areas that are important for both gross motor skills and executive functions [1,25]. Neural networks including the frontal, parietal and motor cortices are not only underlying executive functions, but are also highly involved in gross motor tasks. In addition, the cerebellum and basal ganglia, crucial for motor skills, are also involved in executive functions [1,25–29]. These relations are supported by longitudinal studies showing that better motor skills at baseline are related to better attentional and preparatory processes, which are mainly expressed in the premotor and motor cortex, and the fronto-parietal network, during a working memory task [30]. Also physical activity interventions including coordinative exercises have been shown to enhance brain functioning mainly in the premotor and motor cortex, and in the fronto-parietal network, leading to better interference control [31] and working memory [32].

At a behavioral level, the relation between gross motor skills and executive functions may be explained by the fact that executive functions are involved in motor tasks [2]. Physical activities and complex sports that enhance gross motor skills require focused attention, inhibitory control, and memory of complex sequences, which stimulates the development of executive functions [23,33]. The specificity of the relations between gross motor skills and executive functions may imply that some executive functions are more strongly involved in physical activities or sports that stimulate gross motor skills than other executive functions. However, as shown in the review by van der Fels et al. [3], at the moment it is difficult to draw conclusions about the specific executive functions that are related to gross motor skills due to the scarce number of studies in this field.

### Information processing and lapses of attention

Information processing and attention are cognitive functions that seem to play an important role in the relation between gross motor skills and executive functions. Information processing refers to the efficiency (speed and variability) with which information is processed [34], and develops rapidly during childhood [35–37]. Improvements in information processing have shown to be related to improvements in executive functions [38–40]. Additionally, the consistency of attention is crucial for efficient cognitive performance. Short-term unavailability of attention, also known as lapses of attention, has been shown to affect the speed and quality of cognitive performance [41]. Information processing and attention are also related to gross motor skills in children, although this is mainly investigated in children with developmental disorders [42,43]. It has been shown in studies with typically developing children that relations between gross motor skills and executive functions substantially attenuate when the relations are controlled for processing speed [4,44] or attention/inattention [5,45]. However, no studies have examined the role of information processing and attention together, which was therefore the aim of the current study.

### The present study

The first aim of this study is to systematically investigate relations between gross motor skills and specific aspects of executive functioning (i.e. verbal working memory, visuospatial working memory, response inhibition and interference control) in 8–10 year old typically developing children. The second aim of this study is to investigate the role of information processing (speed and variability) and lapses of attention in the relation between gross motor skills and executive functions. We hypothesize that relations between gross motor skills and specific aspects of executive functions exist, although no explicit hypotheses regarding the specific relations are formulated, because of a lack of previous studies focusing on these. Furthermore, we expect that information processing and lapses of attention are important prerequisites for executive functioning and that relations between gross motor skills and executive functions attenuate after controlling for information processing and lapses of attention. The findings of the present study will be relevant for the screening of children with difficulties in either domain, as problems in one domain (gross motor skills or executive functions) may also imply difficulties in the other domain. Furthermore, a better insight into specific relations between gross motor skills and executive functions will create starting points for gross motor skill interventions that may simultaneously enhance aspects of executive functions.

## Materials and methods

### Participants

Children in this study were part of the “Learning by Moving” project, a large randomized controlled trial assessing the effects of two physical activity interventions on physical and cognitive outcomes, academic achievement, brain structure and brain functioning in primary school children. For the present study, the pretest data on gross motor skills and executive functions were used. In total, 1168 children from grade 3 and grade 4 from 22 regular primary schools were invited to participate. School directors and legal guardians of 891 children (response rate = 83.4%) gave written consent for their children to participate. Children with an estimated IQ < 70 (n = 10) and/or missing values on one of the test scores (n = 149) were excluded (17% of all children with written consent). Full scale IQ was estimated using the Wechsler Intelligence Scale for Children-III subtests block design and information [46]. Reasons for missing test scores were 1) no score for gross motor skills due to absence during testing days at school, injuries, or incorrect test administration (n = 68); 2) no score for information processing and lapses of attention due to absence during testing days or incorrect test administration (n = 13); 3) no response on the parental questionnaire (n = 68). Level of parental education of both parents was requested through a parent-questionnaire and varied from 0 (no education) to 7 (postdoctoral education) [47]. Average educational level of both parents (or from one parent if level of only one parent was specified) was used as a measure of socioeconomic status (SES). The final sample consisted of 732 children (Mean age = 9.16 ± 0.64 years; 50.0% boys; 50.4% 3rd grade; SES = 4.51 ± 1.01). This study was conducted according to the principles expressed in the Declaration of Helsinki and was approved by the Medical Ethical board of the Vrije Universiteit Amsterdam (VCWE-S-15-00197). This study was registered in the Netherlands Trial Register (NL5194).

### Instruments

#### Gross motor skills

Gross motor skills were assessed using three subtests (jumping sideways, moving sideways, and backwards balancing) of the Körper Koordinationstest für Kinder (KTK) [48]. The KTK originally consists of four subtests, but a recent study has shown substantial agreement between three subtests and the original four subtests [49]. Additionally, one item of the Bruininks-Oseretsky Test of Motor Proficiency, Second Edition (BOT-2) was used to measure ball skills [50]. Both test batteries have shown to be reliable and valid for primary school children [48–51].

*Jumping sideways (KTK)*. Children jumped laterally as quickly as possible over a small wooden slat (60 x 4 x 2 cm) for 15 s. The total number of jumps in two trials was used as the score for jumping sideways.

*Moving sideways (KTK)*. Children moved across the floor as quickly as possible in 20 s by stepping on and transferring two plates (25 x 25 x 5.7 cm). Children stepped from the first plate to the next, subsequently lifting and transferring the first plate alongside the second and stepping on it. Each successful transfer from one plate to the next resulted in two points: one for shifting the plate and one for stepping on the next plate. The total number of points on two trials was used as a score for moving sideways.

*Backwards balancing (KTK)*. Children made as many steps backwards as possible on three wooden beams with lengths of 3 m, which were decreasing in width (resp. 6 cm, 4.5 cm, and 3 cm). For each beam, children performed three trials. A maximum of eight steps per trial was counted, resulting in a maximum score of 72 steps.

*Ball skills (BOT-2)*. Ball skills consisted of seven activities (catching a tossed ball with one hand, catching a tossed ball with two hands, dropping and catching a ball with one hand, dropping and catching a ball with two hands, throwing a ball at a target, dribbling a ball with one hand, and dribbling a ball with alternating hands) executed with a tennis ball. Five trials were performed for catching a tossed ball (with one and two hands), dropping and catching a ball (with one and two hands), and throwing a ball at a target. For each correct trial, a child received one point. For dribbling a ball (with one hand and with alternating hands), children had two attempts to dribble 10 times. Based on the highest number of dribbles of the two attempts, a child received a maximum of 7 points. The maximum score for ball skills was 39 points.

#### Executive functions

Executive functions were assessed using the Wechsler Intelligence Scale for Children-III Digit Span subtest and three computer tasks (Visuospatial Working Memory task, Stop Signal task, and Flanker task), performed in E-prime (version 2.0.10.356) on a laptop with a 15.6 inch monitor. All tasks have shown to be valid and reliable in children [52–55].

*Verbal working memory*. Digit Span Backward of the Digit Span task of the WISC-III was used to assess verbal working memory [46]. Children had to verbally reproduce series of digits in reversed order. The test started with two digits and the length of the digit span increased every two trials. When the child was unable to reproduce the two trials of equal length, the test was terminated. For every correct trial, the child received one point. The total score was calculated by multiplying the number of correct trials with the highest length of digit sequence passed [56].

*Visuospatial working memory*. An adapted version of a Visuospatial Memory task was used to assess visuospatial working memory [57,58]. A sequence of yellow circles was shown in a 4 x 4 grid. Children had to recall the reversed order of the sequence and tap the corresponding squares on the computer screen. Level was increased every four trials by increasing the span length. Within a span, there were two sublevels, manipulated by the position of the circles (easy or difficult). The test started with a sequence of two circles. The test was terminated if the child was unable to reproduce the sequence of the two trials within a sublevel. For every correct trial, the child received one point. The total score was calculated by multiplying the number of correct trials with the span of the last correct item. For example, the easy sublevel within two circles gave 2 points and the difficult sublevel within the length of two circles gave 2.5 points [56].

*Response inhibition*. Response inhibition was assessed with the Stop Signal task [55]. The task consisted of go trials (75% of trials) and stop trials (25% of trials). An airplane was presented in every trial, either pointing to the left or to the right. On go trials, children had to press one of two spatially compatible response buttons as quickly as possible after presentation of the airplane. On stop trials, the airplane was followed by a visual stop-signal (i.e. a red traffic-sign displaying ‘STOP’). Children were instructed to inhibit their motor response and not to press a button on stop trials. The stop signal initially appeared 175 ms after the go signal, but was lengthened by 50 ms after correctly inhibited motor responses on stop trials (increasing the difficulty of response inhibition in the next stop trial), and shortened by 50 ms after failure to inhibit the motor response (decreasing the difficulty of response inhibition in the next stop trial). This procedure results in an average success rate at ~50% on stop trials. The task consisted of five blocks; two practice blocks and three experimental blocks. The first practice block consisted of only go trials. The second practice block consisted of 32 trials (25% stop trials). The three experimental blocks each contained 64 trials per block (25% stop trials). Data were analyzed over the three experimental blocks, providing a total of 192 trials. The stop signal reaction time (SSRT) was used as an index for response inhibition and was calculated by subtracting the mean delay between the go and stop signal from the mean reaction time on correct go trials [59].

*Interference control*. A modified version of the Flanker task was used to assess interference control [60]. The target stimulus was an arrow pointing to the left or to the right, and children were instructed to press the compatible button as quickly as possible. The task consisted of neutral trials, congruent trials, or incongruent trials. In neutral trials, the arrow was flanked on the left and right side with two horizontal lines. In the congruent trials, the arrow was flanked on the left and right side with two identical arrows, pointing to the same side as the target arrow. In incongruent trials, the arrow was flanked on the left and right side with two identical arrows pointing in the opposite direction. The task consisted of one practice block with 24 trials and three experimental blocks with 72 trials, with equal probabilities of neutral, congruent or incongruent trials. The difference in mean reaction time on congruent and incongruent trials was used as a measure for interference control.

#### Information processing and lapses of attention

Information processing and lapses of attention were assessed from the correct go trials of the Stop Signal task. An ex-Gaussian model was applied to calculate the parameters. This model combines a normal distribution shape of individual reaction times with an exponential component on the right side of the distribution [61]. The model determines average processing speed and variability (measured by the mean and standard deviation of the normal distribution component of the Ex-Gaussian curve), corrected for extremely slow responses, or lapses of attention (measured by the mean of the exponential component). Ex-Gaussian analysis was performed in MATLAB (2018a) [61]. Relevance of the ex-Gaussian distribution is proved in previous studies [62,63].

### Procedure

Children were tested on their gross motor skills and cognitive functions by trained examiners using standardized protocols within a two-week period. Gross motor skills were assessed during one or two (depending on the class size) physical education lessons in circuit form with tests administered in a random order. Executive function tasks were assessed in a quiet room at children’s own school. Children were individually tested by trained examiners. The tests were assessed in two parts on two days. The Stop Signal task and the Digit Span task were assessed on the first day. The Flanker task and the Visuospatial Working Memory task were assessed on the second day.

### Data analysis

Initial analyses were performed in IBM SPSS Statistics version 25.0. Outliers (z ≤ -3.29 or ≥ 3.29) were replaced with a value one unit greater than the next non-outlier value [64]. Z-scores were calculated for the four gross motor skill tests, verbal working memory, visuospatial working memory, response inhibition, interference control, and for processing speed, processing variability, lapses of attention, age, and SES. A lower z-score indicated a better score for response inhibition, interference control, processing speed, processing variability and lapses of attention and these scores were therefore transformed so that a higher score indicated better performance for all variables. These (transformed) z-scores were used for all analyses.

A principal component analysis on the z-scores of the gross motor skill tests was performed to reduce the gross motor variables into one factor explaining most of the shared variance of the four gross motor skill tests. It was expected that all four motor skill tests loaded highly onto one factor (gross motor skills). The (Bartlett) factor score(s) was used for all analyses.

Pearson correlations were calculated to obtain raw correlations between all study variables. A multivariate multilevel regression analysis (MLwiN, version 3.01) was performed for the main analysis in order to take into account the variability between classes and nesting of children within the classes. Verbal working memory, visuospatial working memory, response inhibition and interference control were used as dependent variables in the models (level 1). A random intercept was included for each class (level 3). Three models were built. Model 1 contained only covariates (grade, age, sex, SES) as predictors of the dependent variables. Those covariates were included, because they have previously shown to attenuate relations between gross motor skills and executive functions in children [2,5,44,45]. IQ was not included as a covariate in the analysis, because there is substantial construct overlap between IQ and executive functioning [65–67]. In Model 2, gross motor skills were added to investigate the contribution of gross motor skills to the executive function tasks. In Model 3, processing speed, variability and lapses of attention were added to investigate the role of information processing and lapses of attention and to investigate the relation between gross motor skills and executive functions after controlling for information processing and lapses of attention. The model fit was evaluated by comparing the deviance (-2*log-likelihood) of the first model to the second model and of the second to the third model using a χ^2^ difference test. If the model was significantly better than the previous model, it was investigated per dependent variable whether predictors were significant in this model. Level of significance was set at p < 0.05 (two-sided). Effect sizes (ESs) were calculated as (estimated effect of predictor B * 2) / √(1 –B^2^) [68]. An effect size below 0.20 is considered negligible, 0.20–0.49 small, 0.50–0.79 medium and above 0.80 strong [69].

## Results

The raw scores on the gross motor and executive function tests are shown in Table 1. S1 Table shows the correlation matrix and the factor loadings of the principal component analysis for gross motor skills. The four gross motor skill components loaded highly (> 0.6) onto one factor and explained 48.2% of the total variance. S2 Table shows the raw correlations between all study variables.

**Table 1:** Raw test scores of the study population (n = 732).

Variable	Mean (SD)
*Gross motor skills*	
Jumping sideways (total score)	48.86 (15.71)
Moving sideways (total score)	34.20 (9.14)
Backwards balancing (total score)	40.69 (13.67)
Ball skills (total score)	30.80 (5.17)
*Executive functions*	
Verbal working memory (total score)	14.62 (8.60)
Visuospatial working memory (total score)	47.53 (22.77)
Response inhibition (ms)	250.03 (49.16)
Interference control (ms)	130.62 (63.04)
*Information processing and lapses of attention*	
Speed of information processing (ms)	506.54 (93.17)
Variability of information processing (ms)	87.83 (22.45)
Lapses of attention (ms)	125.70 (41.99)

Table 2 shows the results of the multivariate multilevel regression models. Model 2 was significantly better than model 1, Δχ^2^(4) = 58.09, p < 0.001. When the univariate contribution of gross motor skills to the four executive functions in Model 2 was investigated, it was shown that gross motor skills were significantly related to verbal working memory, t = 2.44, p = 0.015, ES = 0.19, visuospatial working memory, t = 6.42, p < 0.001, ES = 0.50, and response inhibition, t = 5.00, p < 0.001, ES = 0.40, indicating that better gross motor skills are related to better verbal working memory (negligible effect), visuospatial working memory (medium effect) and response inhibition (small effect). Gross motor skills were not significantly related to interference control.

**Table 2:** Results of the multivariate multilevel regression analysis (n = 732).

	Model 1			Model 2			Model 3		
	B	SE	p	B	SE	p	B	SE	p
*Verbal working memory*									
Random intercept	-0.385	0.094	<0.001	-0.371	0.098	<0.001	-0.356	0.097	<0.001
Grade^a^	0.623	0.140	<0.001	0.579	0.145	<0.001	0.555	0.144	<0.001
Age	-0.140	0.056	0.012	-0.164	0.056	0.003	-0.155	0.056	0.006
Sex^b^	0.181	0.071	0.010	0.193	0.070	0.006	0.188	0.070	0.008
SES^c^	0.121	0.038	0.001	0.122	0.038	0.001	0.116	0.038	0.002
Gross motor skills				0.095	0.039	**0.015**	0.077	0.040	0.053
Processing speed							0.059	0.059	0.320
Processing variability							0.016	0.060	0.787
Lapses of attention							0.097	0.042	**0.022**
Variance classes^d^	0.089	0.030	0.003	0.104	0.034	0.002	0.098	0.032	0.002
Variance children^d^	0.841	0.045	<0.001	0.831	0.045	<0.001	0.829	0.045	<0.001
*Visuospatial working memory*									
Random intercept	-0.189	0.074	0.010	-0.149	0.073	0.041	-0.128	0.074	0.085
Grade^a^	0.471	0.111	<0.001	0.351	0.111	0.002	0.320	0.113	0.005
Age	-0.087	0.056	0.123	-0.132	0.055	0.017	-0.120	0.055	0.030
Sex^b^	-0.007	0.073	0.926	0.024	0.071	0.733	0.003	0.071	0.966
SES^c^	0.139	0.038	<0.001	0.128	0.037	0.001	0.116	0.037	0.002
Gross motor skills				0.244	0.038	**<0.001**	0.224	0.039	**<0.001**
Processing speed							-0.105	0.060	0.081
Processing variability							0.206	0.061	**0.001**
Lapses of attention							0.132	0.042	**0.002**
Variance classes	0.006	0.013	0.644	0.008	0.013		0.012	0.014	0.392
Variance children	0.941	0.051	<0.001	0.896	0.048	<0.001	0.872	0.047	<0.001
*Response inhibition*									
Random intercept	-0.267	0.075	<0.001	-0.237	0.073	0.001	-0.222	0.070	0.001
Grade^a^	0.327	0.113	0.004	0.233	0.111	0.036	0.206	0.107	0.054
Age	0.068	0.056	0.226	0.032	0.056	0.565	0.046	0.054	0.393
Sex^b^	0.156	0.072	0.030	0.181	0.071	0.011	0.167	0.069	0.015
SES^c^	0.056	0.038	0.137	0.046	0.037	0.214	0.023	0.036	0.512
Gross motor skills				0.195	0.039	**<0.001**	0.132	0.038	**<0.001**
Processing speed							0.003	0.058	0.958
Processing variability							0.041	0.059	0.486
Lapses of attention							0.290	0.041	**<0.001**
Variance classes	0.013	0.014	0.353	0.008	0.013	0.538	0.005	0.012	0.676
Variance children	0.918	0.049	<0.001	0.893	0.048	<0.001	0.830	0.045	<0.001
*Interference control*									
Random intercept	-0.186	0.072	0.009	-0.181	0.073	0.013	-0.174	0.073	0.017
Grade^a^	0.399	0.108	<0.001	0.387	0.111	0.001	0.377	0.112	0.001
Age	0.031	0.055	0.576	0.025	0.056	0.656	0.031	0.056	0.583
Sex^b^	0.053	0.071	0.455	0.056	0.071	0.435	0.045	0.071	0.527
SES^c^	0.039	0.037	0.287	0.041	0.037	0.271	0.029	0.037	0.434
Gross motor skills				0.023	0.039	0.557	-0.004	0.040	0.927
Processing speed							-0.045	0.060	0.451
Processing variability							0.056	0.061	0.352
Lapses of attention							0.127	0.042	**0.003**
Variance classes	0.007	0.013	0.590	0.008	0.013	0.538	0.009	0.013	0.489
Variance children	0.885	0.048	<0.001	0.885	0.048	<0.001	0.872	0.047	<0.001
Deviance	7910.333			7852.241			7765.889		

*Note*. The model for visuospatial working memory was based on 723 children, because of missing values for nine children; the model for interference control was based on 719 children, because of missing values for thirteen children;

^a^Grade 3 was the reference category;

^b^Boys was the reference category;

^c^Socioeconomic status;

^d^These values represent respectively the between and within class variance; significant predictors related to the research questions are shown in **bold**.

The addition of information processing and lapses of attention in Model 3 did significantly improve the model, Δχ^2^(4) = 86.35, p < 0.001. The influence of lapses of attention was significantly related to verbal working memory, t = 2.31, p = 0.022, ES = 0.19, visuospatial working memory, t = 3.14, p = 0.002, ES = 0.27, response inhibition, t = 7.07, p < 0.001, ES = 0.61, and interference control, t = 3.02, p = 0.003, ES = 0.26. Less variability in processing speed was related to better visuospatial working memory, t = 3.38, p = 0.001, ES = 0.42, but not to verbal working memory, response inhibition and interference control. Processing speed did not show significant relations with the executive function tasks. Thus, children with less influence of lapses of attention perform better on verbal working memory (negligible effect), visuospatial working memory (small effect), response inhibition (medium effect) and interference control (small effect) and children with less variability in processing speed performed better on visuospatial working memory (small effect).

After controlling for information processing and lapses of attention, the significant relations between gross motor skills and visuospatial working memory, t = 5.74, p < 0.001, ES = 0.46, and between gross motor skills and response inhibition, t = 3.47, p < 0.001, ES = 0.27, did attenuate. The relation between gross motor skills and verbal working memory did not remain significant. Thus, after controlling for information processing and lapses of attention, gross motor skills were only significantly related to visuospatial working memory (small effect) and response inhibition (small effect).

## Discussion

This study aimed to investigate relations between gross motor skills and specific aspects of executive functions (i.e. verbal working memory, visuospatial working memory, response inhibition and interference control). Additionally, the role of information processing (speed and variability) and lapses of attention in the relation between gross motor skills and executive functions were examined. This study confirmed previous findings of relations between gross motor skills and specific aspects of executive functions. This study extends existing knowledge by showing that gross motor skills were significantly related to verbal working memory, visuospatial working memory and response inhibition. However, after controlling for information processing and lapses of attention, the relation between gross motor skills and visuospatial working memory and response inhibition attenuated, although remaining significant (small effect). The relation between gross motor skills and verbal working memory on the other hand became non-significant. Furthermore, lapses of attention was a significant predictor for verbal working memory (although the effect was negligible), visuospatial working memory (small effect), response inhibition (medium effect) and interference control (small effect), whereas processing speed did not predict any of the executive function tasks. Variability in processing speed was only a significant predictor for visuospatial working memory (small effect).

### Neuropsychological explanations

The relations between gross motor skills and executive functions found in this study can be explained using a neuropsychological framework, stating that there is an overlap in neural networks that are important for both gross motor skills and executive functions [1,25]. From this perspective, our results indicate that the neural network supporting gross motor skills may share more overlap with the neural network supporting visuospatial working memory and response inhibition than with the neural network supporting verbal working memory and interference control. To confirm this underlying neural mechanism, studies are needed that investigate the neural networks related to gross motor skills and specific executive functions.

### Explanations at a behavioral level

Besides the neuropsychological framework, the relations between gross motor skills and executive functions found here can be explained at a behavioral level [70]. Gross motor skills and executive functions are required for and trained during regular physical activity and cognitively challenging sports [24,71], as goal-directed behavior is needed during these sports and activities to adapt to a constantly changing environment. Therefore, involvement in sports not only improves gross motor skills, but also stimulates the development of executive functions [70,72]. It has been shown that visuospatial working memory is involved in the planning and control of movements [73–75]. For example, to perform a gross motor task such as catching a ball or balancing through the environment, visuospatial information about the ball, the environment and the body position is needed to perform the task [76]. In contrast, the verbal system is more important for verbal information and is therefore less involved in the planning and control of movement [77]. This can explain the finding that gross motor skills are related to visuospatial working memory, but not to verbal working memory, as was also shown by Rigoli et al. [19] and Alesi et al. [23].

Furthermore, response inhibition is needed in dynamic sports, where movements have to be continually adapted to the constantly changing environment, which requires constant inhibition of initial motor actions, and adaption and updating of motor actions [23]. This explains why we found a relation between gross motor skills and response inhibition. No relation between gross motor skills and interference control was found. As the definition of interference control (the ability to cognitively compress conflicting stimuli) already implies a higher involvement of cognition compared to response inhibition, our results suggest that gross motor skills are more strongly related to aspects of executive functions that are most directly involved in, and share common underlying processes with, gross motor skills.

### The role of information processing and lapses of attention

Lapses of attention was a significant predictor for verbal working memory, visuospatial working memory, response inhibition and interference control, thereby confirming our hypothesis. Variability in processing speed (as corrected for lapses of attention) only predicted visuospatial working memory, while processing speed was not related to the investigated aspects of executive functions. These results indicate that lapses of attention, are more strongly related to performance on an executive function task than the mean reaction time and/or variability in reaction time, after controlling for lapses of attention. Our findings support the theory of the *worst performance rule*, which states that in multi-trial tasks, worst performance trials (e.g. slowest reaction times, indicating lapses of attention) are stronger predictors of cognitive performance than processing speed and variability [41,78,79]. Maintaining attention thus seems extremely important for fast and accurate task performance. This underlines the importance of taking into account lapses of attention rather than processing speed and/or variability when investigating the relation between gross motor skills and executive functions.

### Strengths and limitations

The strengths of this study include the large sample of typically developing children that was examined, making it likely that the results are generalizable to 8–10 year old children. Additionally, the four executive function tasks that were used, and the inclusion of an extensive approach to analyze information processing and lapses of attention resulted in a deeper insight into the specificity of the relation between gross motor skills and executive functions, and the role of information processing and lapses of attention within this relation.

However, there are also some limitations. We used a cross-sectional design, which makes it impossible to make statements about the causal relations between gross motor skills and executive functions. Therefore, intervention studies are necessary to investigate whether a similar pattern of specific (causal) relations between gross motor skills and executive functions can be found. Furthermore, the Stop Signal task that was used to assess information processing and lapses of attentions was also used to measure response inhibition. Therefore, the inhibition component could not completely been separated from information processing and lapses of attention. For future studies, it is recommended to use different tests for response inhibition and information processing and lapses of attention.

Our results on the specific relations between gross motor skills and executive functions can be used for the development of cognitively engaging interventions. Only a few studies have examined the effects of this type of intervention, in which children are cognitively engaged through motor demanding tasks [80]. Our results suggest that training of gross motor skills may contribute to the development of specific executive functions, e.g. visuospatial working memory and response inhibition in children. Furthermore, our results propose that interventions targeting lapses of attention, may subsequently improve executive functions.

### Conclusions

In conclusion, after controlling for information processing and lapses of attention, gross motor skills are specifically related to visuospatial working memory and response inhibition. Additionally, lapses of attention were related to all executive functions, whereas processing speed was not. Variability in processing speed was only related to visuospatial working memory. The results indicate that gross motor skills are related to aspects of executive functions that are most directly involved in, and share common underlying processes with, gross motor skills. Furthermore, the results underline the importance of taking into account lapses of attention rather than information processing (speed and variability), when investigating the relation between gross motor skills and executive functions.

## Supporting information

S1 Table Correlation matrix and factor loadings for the principal component analysis for gross motor skills. (DOCX)

S2 Table Pearson correlations between the study variables (n = 732). (DOCX)
