# Supplementary material for: Relations between gross motor skills and executive functions, controlling for the role of information processing and lapses of attention in 8-10 year old children
Source: PLoS One. 2019 Oct 24;14(10):e0224219. doi: 10.1371/journal.pone.0224219 (PMC6812776; doi:10.1371/journal.pone.0224219)
Supplement: S1 Table — (DOCX) [file pone.0224219.s001.docx]

**S1 Table. Correlation matrix and factor loadings for the principal component analysis for gross motor skills.**

|  | Jumping sideways (total number of jumps) | Moving sideways (total points) | Backwards balancing (total steps) | Ball skills (total points) | Factor loading |
| --- | --- | --- | --- | --- | --- |
| Jumping sideways | 1 |  |  |  | 0.709 |
| Moving sideways | 0.428 | 1 |  |  | 0.798 |
| Backwards balancing | 0.342 | 0.260 | 1 |  | 0.624 |
| Ball skills | 0.357 | 0.246 | 0.196 | 1 | 0.631 |
