# Supplementary material for: Relations between gross motor skills and executive functions, controlling for the role of information processing and lapses of attention in 8-10 year old children
Source: PLoS One. 2019 Oct 24;14(10):e0224219. doi: 10.1371/journal.pone.0224219 (PMC6812776; doi:10.1371/journal.pone.0224219)
Supplement: S2 Table — (DOCX) [file pone.0224219.s002.docx]

**S2 Table. Pearson correlations between the study variables (n = 732).**

|  | Gross motor skills | Executive functions | | | | Information processing and lapses of attention | | | Sociodemographic variables | | |
| --- | --- | --- | --- | --- | --- | --- | --- | --- | --- | --- | --- |
|  |  | Verbal working memory | Visuospatial working memory | Response inhibition (ms) | Interference control (ms) | Processing speed (ms) | Processing variability (ms) | Lapses of attention (ms) | Age (years) | Sex | SES |
|  |  |  | | | |  | | |  | | |
| Motor skills^a^ | 1 |  |  |  |  |  |  |  |  |  |  |
| Verbal working memory^b^ | .08* | 1 |  |  |  |  |  |  |  |  |  |
| Visuospatial working memory^b^ | .25** | .22** | 1 |  |  |  |  |  |  |  |  |
| Response inhibition (ms)^c^ | .25** | .14** | .13** | 1 |  |  |  |  |  |  |  |
| Interference control (ms)^c^ | .10** | .14** | .17** | .08* | 1 |  |  |  |  |  |  |
| Processing speed (ms)^c^ | -.06 | .05 | -.01 | -.10** | -.05 | 1 |  |  |  |  |  |
| Processing variability (ms)^c^ | -.09* | .05 | .06 | -.10** | -.03 | .81** | 1 |  |  |  |  |
| Lapses of attention (ms)^c^ | .23** | .09* | .15** | .32** | .15** | -.49** | -.47** | 1 |  |  |  |
| Age (years) | .37** | .05 | .05 | .18** | .17** | .04 | -.02 | .06 | 1 |  |  |
| Sex | -.08* | .11** | .00 | .08* | -.04 | .02 | .05 | .01 | -.06 | 1 |  |
| SES^d^ | .01 | .17** | .16** | .05 | -.05 | .00 | .03 | .09* | -.16** | -.01 | 1 |

*Notes.* Pearson correlation calculated on (transformed) z-scores. ^a^Bartlett factor score calculated from the standardized scores of the four motor skill tests; ^b^Total score, calculated by multiplying the number of correct trials with the highest length of digit sequence passed; ^d^Socioeconomic Status; * p < 0.05; ** p < 0.01.
